# *Bacopa monnieri* (L.) Wettst. Extract Improves Memory Performance via Promotion of Neurogenesis in the Hippocampal Dentate Gyrus of Adolescent Mice

**DOI:** 10.3390/ijms21093365

**Published:** 2020-05-09

**Authors:** Hang Thi Nguyet Pham, Hong Nguyen Tran, Phuong Thi Nguyen, Xoan Thi Le, Khoi Minh Nguyen, Sinh Viet Phan, Masanori Yoneyama, Kiyokazu Ogita, Taro Yamaguchi, William R. Folk, Masamitsu Yamaguchi, Kinzo Matsumoto

**Affiliations:** 1 Department of Pharmacology and Biochemistry, National Institute of Medicinal Materials, Hoan Kiem District, Hanoi 100000, Vietnam; trannguyenhong91@gmail.com (H.N.T.); phuongduocly@gmail.com (P.T.N.); xoanle@nimm.org.vn (X.T.L.); khoi_nguyenminh@yahoo.co.uk (K.M.N.); 2 National Geriatric Hospital, Dong Da District, Hanoi 100000, Vietnam; dssinhbm@yahoo.com; 3 Faculty of Pharmaceutical Sciences, Setsunan University, Osaka 573-0101, Japan; m-yone@pharm.setsunan.ac.jp (M.Y.); ogita@pharm.setsunan.ac.jp (K.O.); t-yamagu@pharm.setsunan.ac.jp (T.Y.); 4 Department of Biochemistry, University of Missouri, Columbia, MO 65211, USA; FolkW@missouri.edu; 5 Department of Applied Biology, Kyoto Institute of Technology, Matsugasaki, Sakyo-ku, Kyoto 603-8585, Japan; myamaguc@kit.ac.jp; 6 Department of Kampo Pharmacology, Daiichi University of Pharmacy, Fukuoka 815-8511, Japan

**Keywords:** *Bacopa monnieri*, cognitive improvement, neurogenesis, neural stem/progenitor cell, dentate gyrus, RNAseq

## Abstract

*Bacopa monnieri* L. Wettst. (BM) is a botanical component of Ayurvedic medicines and of dietary supplements used worldwide for cognitive health and function. We previously reported that administration of BM alcoholic extract (BME) prevents trimethyltin (TMT)-induced cognitive deficits and hippocampal cell damage and promotes TMT-induced hippocampal neurogenesis. In this study, we demonstrate that administration of BME improves spatial working memory in adolescent (5-week- old) healthy mice but not adult (8-week-old) mice. Moreover, improved spatial working memory was retained even at 4 weeks after terminating 1-week treatment of adolescent mice. One-week BME treatment of adolescent mice significantly enhanced hippocampal BrdU incorporation and expression of genes involved in neurogenesis determined by RNAseq analysis. Cell death, as detected by histochemistry, appeared not to be significant. A significant increase in neurogenesis was observed in the dentate gyrus region 4 weeks after terminating 1-week treatment of adolescent mice with BME. Bacopaside I, an active component of BME, promoted the proliferation of neural progenitor cells in vitro in a concentration-dependent manner via the facilitation of the Akt and ERK1/2 signaling. These results suggest that BME enhances spatial working memory in healthy adolescent mice by promoting hippocampal neurogenesis and that the effects of BME are due, in significant amounts, to bacopaside I.

## 1. Introduction

The capacity of neural stem cells to proliferate and differentiate into neuronal cells in the brain contributes to prevention and recovery from memory loss and other neurodegenerative diseases [1,2]. In rodents, neurogenesis has been experimentally demonstrated in two specific regions of the brain: the sub-ventricular zone (SVZ) and dentate gyrus of the hippocampus [2,3]. The dentate gyrus is a vital part of the hippocampus with the ability to self-renew in young rodents, although whether this occurs in adults is unclear. Neurogenesis in the dentate gyrus region plays a significant role in hippocampus-dependent learning and memory [4,5], and depletion of newly regenerated cells in this region leads to brain damage [6]. Promotion of neurogenesis in the hippocampal dentate gyrus region by enhancing the self-regenerative ability of neuronal stem cells offers potential for maintaining and improving cognitive function [7].

*Bacopa monnieri* (L.) Wettst. (BM) is a medicinal plant traditionally used as a neural tonic to improve impaired cognitive function and promote longevity [8,9]. Recent evidence demonstrates that BM extracts (BME) possess neuroprotective effects against brain dysfunction, such as epilepsy, cognitive deficits in animal models and cerebral ischemia [8,10,11,12,13,14]. In previous studies, we reported that BME made with alcohols, containing 21.8% bacoside A and 11.0% bacopaside I, attenuated transient cerebral ischemia-induced cognitive deficits. Our studies suggested that bacopaside I, a major triterpenoid component of BME, has a significant role in the effect of BME, since it protected neuronal cells from oxygen- and glucose-deprivation-induced damage via protein kinase C (PKC) and phosphatidylinositol-3 kinase (PI3K)/Akt mechanisms in organotypic hippocampal slice cultures [8]. In addition, we recently reported that BME ameliorates trimethyltin (TMT)-induced cognition dysfunction by protecting hippocampal neurons from TMT-induced lesions and partly by promoting neurogeneration in the hippocampal dentate gyrus region [15]. These results prompted us to hypothesize that BME may have the potential to improve cognitive function in healthy animals by promoting neurogenesis in the hippocampus. To test this hypothesis, we have examined whether systemic administration of BME improves cognitive function in healthy animals of different ages (adolescent and adult), as the number, density, and proportion of neuronal and non-neuronal cells dramatically changes between adolescent and adult rodents [16,17]. We measured changes in gene expression and replication in the hippocampus, and also evaluated the effects of bacopaside I on neurogenesis, using an in-vitro neural stem cell culture system. The results demonstrate that BME enhancement of cognitive performance is accompanied by promotion of neurogenesis in the hippocampal dentate gyrus of adolescent mice, but not adult mice.

## 2. Results

### 2.1. Effect of BME on Spatial Working Memory in Adolescent (5-Week-Old) and Adult (8-Week-Old) Mice after 7- and 28-Day Treatment

**Figure 1:**
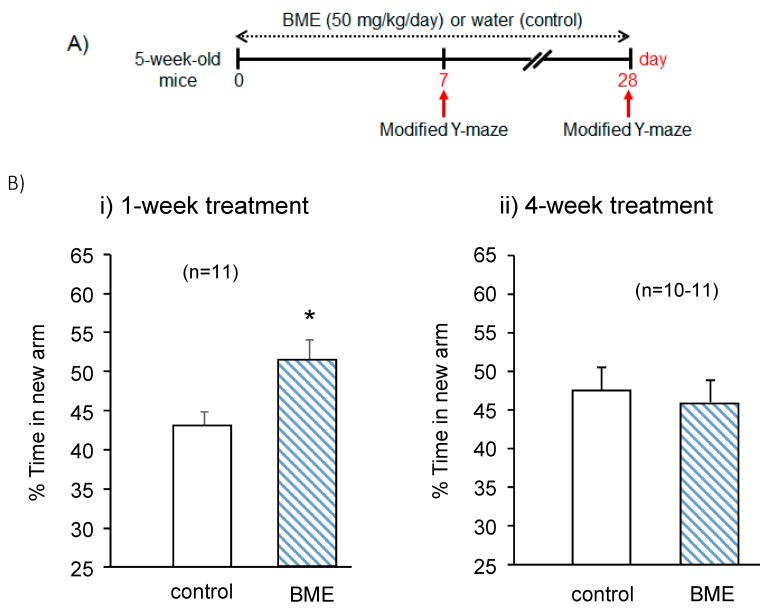
Effect of BME (50 mg/kg) on spatial working memory in adolescent mice after 7- and 28-day treatment. Spatial working memory was elucidated by the modified Y-maze test. Sample and test trials were conducted for 5 min with a 30-min interval (**A**). In the sample trial, each mouse was individually placed in the maze with one of the three arms closed. The arm that was closed in the sample trial was defined as the new arm in the test trial. Summarized data was obtained on days 7 and 28 after the BME treatment (**B**). Results are expressed as percent time animals spent exploring the new arm in the test trial. Each data column represents the mean ± SEM (*n* = 10–11). * *p* < 0.05 vs. the control group.

To evaluate the effect of BME treatment on the spatial cognitive performance of normal mice, we conducted a modified Y maze test on days 7 and 28 after starting daily administration of BME with adolescent mice. As shown in Figure 1B, for control and BME groups, the ratio of times spent visiting the novel arm to the random chance level of 33.3% to enter to this arm indicates a preference for the novel arm over the other familiar arms. This index of time spent in a new arm represents spatial working memory [10,15,18]. The fraction of time spent exploring the novel arm was significantly greater in the animals treated with BME for 7 days than for those treated with vehicle water for the same period (t(18) = −2.635, *p* = 0.017, water-treated group vs. BME-treated group). However, when spatial working memory of the same animal groups was determined on day 28 after consecutive daily administration of BME, no significant difference was observed between the BME-treated group and vehicle-treated group (t(19) = 0.443, *p* = 0.663, water-treated group vs. BME-treated group).

**Figure 2:**
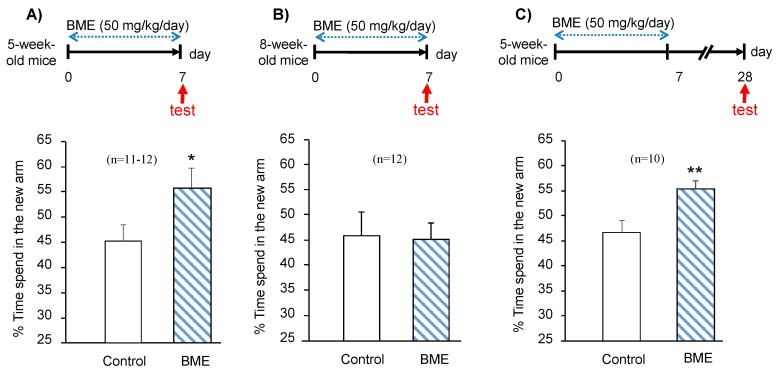
Effect of BME (50 mg/kg) on spatial working memory in 5-week-old and 8-week-old mice after 7 days of treatment. Spatial working memory was elucidated by the modified Y-maze test. Red arrows indicate the day behavioral tests were conducted. Summarized data obtained on 5-week-old mice (**A**) and 8-week-old mice (**B**) after 7 days BME treatment; 5-week-old mice received daily BME at a dose of 50 mg/g (p.o.) for 7 days, and then the spatial working memory was evaluated at 28 days after the last drug administration (**C**). Results are expressed as % of the time animals spent exploring the novel arm in the test trial. Each data column represents the mean ± SEM (*n* = 10–12). ** *p* < 0.01; * *p* < 0.05 vs. the control group.

Next, we examined whether BME-enhancement of spatial working memory depends on the administration period of BME (1–4 weeks) or the age of animals (adolescent or adult) or both. We started a 7-day period of BME (50 mg/kg/day, p.o.) administration with adolescent animals (5-week-old) or adult animals (8-week-old) and then evaluated spatial working memory. Administration of BME significantly enhanced the spatial working memory of the adolescent animals (t(21) = −2.359, *p* = 0.028, water-treated adolescent group vs. BME-treated adolescent group) but not the adult animals (t(22) = 0.253, *p* = 0.803, water-treated adult group vs. BME-treated adult group) (Figure 2A,B) The enhancement of spatial working memory in the adolescent animals who received 7-day BME treatment persisted through day 28 (t(18) = −3.012, *p* = 0.007, water-treated group vs. BME-treated group) (Figure 2C).

We also examined whether the effect of BME administration for 7 days occurs in adult mice using the same paradigm. As shown in Figure 2B, no significant difference between water and BME-treated mice in the spatial working memory was detected, indicating that the enhancing effect of BME upon spatial working memory occurs only in the animals which received BME administration during adolescence.

### 2.2. Determination of BrdU(+) Cells and Differentiation of BrdU(+) Cells to Mature Neurons in the Hippocampal Dentate Gyrus of Adolescent Mice

**Figure 3:**
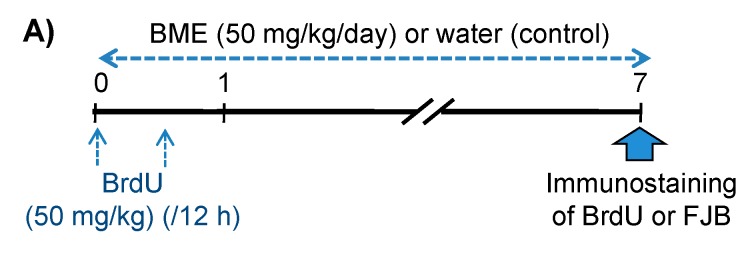

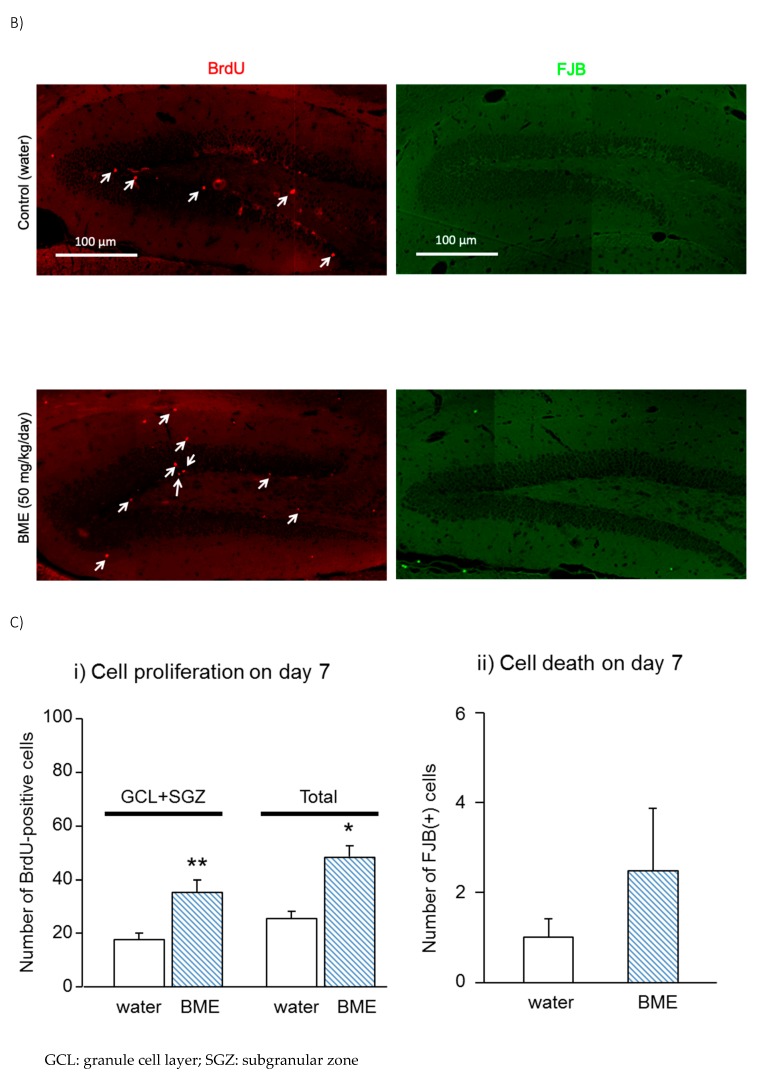
Effects of BME (50 mg/kg) on FJB-positive cells and BrdU incorporation in 5-week-old mice. (**A**) Experimental schedule. Animals were given either BME (50 mg/kg, p.o) or water for 7 consecutive days, and all animals received BrdU (50 mg/kg, i.p.) with a 12-h interval on the first day of the experiment and were then decapitated 1 h after BME treatment on day 7. The sagittal hippocampal sections were then immunostained with antibodies for FJB and BrdU. (**B**) Typical fluorescence micrographs of immunostaining for FJB and BrdU in the hippocampal dentate gyrus of mice treated with BME or water. White arrows indicate the BrdU-positive cells. (**C**) Summarized data on cell proliferation in the total dentate gyrus region and sub-regions (GCL ± SGZ) and cell death in the hippocampal dentate gyrus analyzed on day 7. Each data column represents the mean ± SEM, calculated from four animals in each group. ** *p* < 0.01; * *p* < 0.05 compared to water-treated group at the same area of the mice hippocampus.

Neurogenesis in the hippocampal dentate gyrus reportedly plays an important role in cognitive performance [19]. Based on the results described above, daily administration of BME (50 mg/kg/day, p.o.) to adolescent mice was conducted from day 0 for seven days to examine if BME had an impact on the hippocampal neurogenesis in adolescent animals. Newly generated cells and dying neuronal cells in the hippocampal dentate gyrus regions were detected as 5-Bromo-2′-Deoxyuridine (BrdU)- and Fluoro-Jade B (FJB)-positive cells, respectively, on day 7 (Figure 3A). As shown in Figure 3B,C, BME treatment significantly increased the number of BrdU-positive cells in the granular cell layer (GCL) and subgranular zone of the dentate gyrus (water-treated group vs. BME-treated group, *p* < 0.01) as well as the total number of BrdU-positive cells in the whole dentate gyrus region (water-treated group vs. BME-treated group, *p* < 0.05). However, BME treatment had no effect on the number of FJB-positive cells in the hippocampal region (Figure 3C).

**Figure 4:**
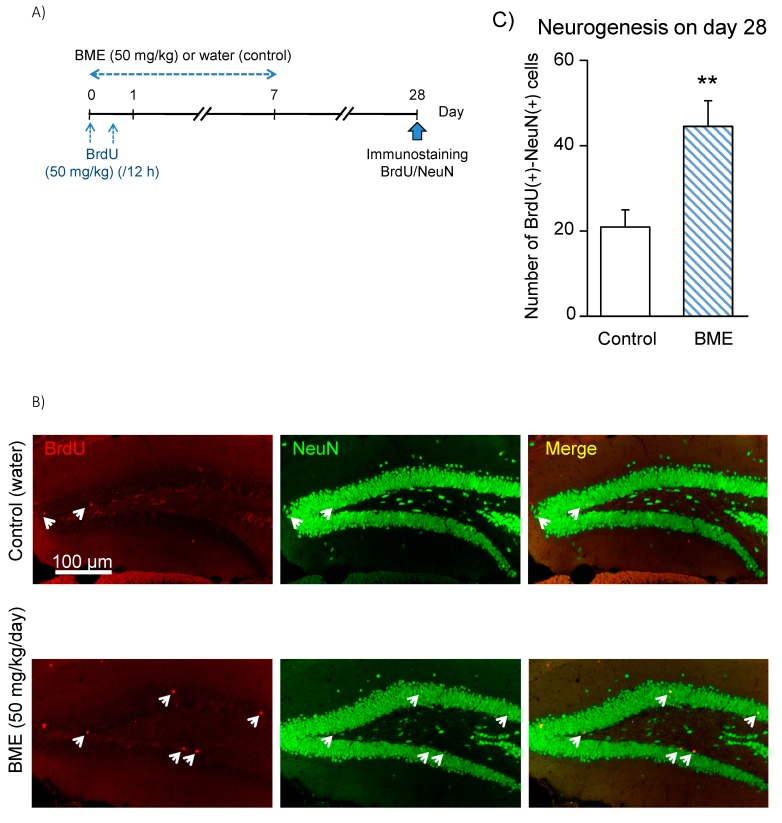
Effect of BME on neuronal differentiation of BrdU(+) cells in 5-week-old mice. (**A**) Experimental schedule. Animals were given either BME (50 mg/kg, p.o) or water for 7 consecutive days, and all animals received BrdU (50 mg/kg, i.p.) with a 12-h interval on the first day of the experiment and were then decapitated on day 28 post-treatment to prepare sagittal hippocampal sections. The sections were immunostained with antibodies for BrdU and NeuN. (**B**) Fluorescence micrographs of NeuN(+) cells (green) and BrdU(+) cells (red) in the dentate gyrus of the water- and BME-treated groups. White arrows indicate BrdU-NeuN double-positive cells. (**C**) Neurogenesis detected in the GCL ± SGZ of dentate gyrus on day 28 after 7-day treatment with BME. Each data column represents the mean ± SEM, calculated from four animals in each group. ** *p* < 0.01 compared to water-treated group.

Moreover, to assess the fate of cells in the dentate gyrus 3 weeks after terminating the BME administration, we analyzed the increase in matured neuronal cells, which are immune-positive to both BrdU and neuronal nuclei (NeuN), as an index of neurogenesis on day 28 in the GCL ± SGZ of the dentate gyrus (Figure 4). The increase in newly generated neuronal cells was about two times more in the animals pretreated with BME during adolescence (5 to 6 weeks old) than in the animals pretreated with vehicle water during the same periods (water-treated group vs. BME-treated group, *p* < 0.01).

### 2.3. BME Treatment-Induced Changes Gene Expression in the Hippocampus

We conducted RNASeq analysis to investigate how the treatment of adolescent animals with BME (50 mg/kg) for 7 days affects the expression of hippocampal genes. Treatment with BME (50 mg/kg) of adolescent mice induced at least two-fold changes in the expression levels of 1357 genes (*p* < 0.05), of which 29 genes were categorized as genes involved in “neurogenesis and/or memory” by Gene Functional Annotation (Table S1). Genes for neuronal signaling pathways for neurogenesis, cell survival and replication, including neurotrophin receptors (NTRK2, NTRK3) and several others identified in previous studies, such as phosphoinositide 3-kinases/protein kinase B (PI3K-Ak), cAMP response element-binding protein (CREB), and cholinergic receptors whose expressions were affected by BME [8,10] were identified by RNAseq using a cutoff value of multimodal *p* < 0.05 and fold-change > 1.2 or < −1.2 (Table 1).

### 2.4. Bacopaside I Enhances Proliferation of Neural Progenitor Cells

**Figure 5:**
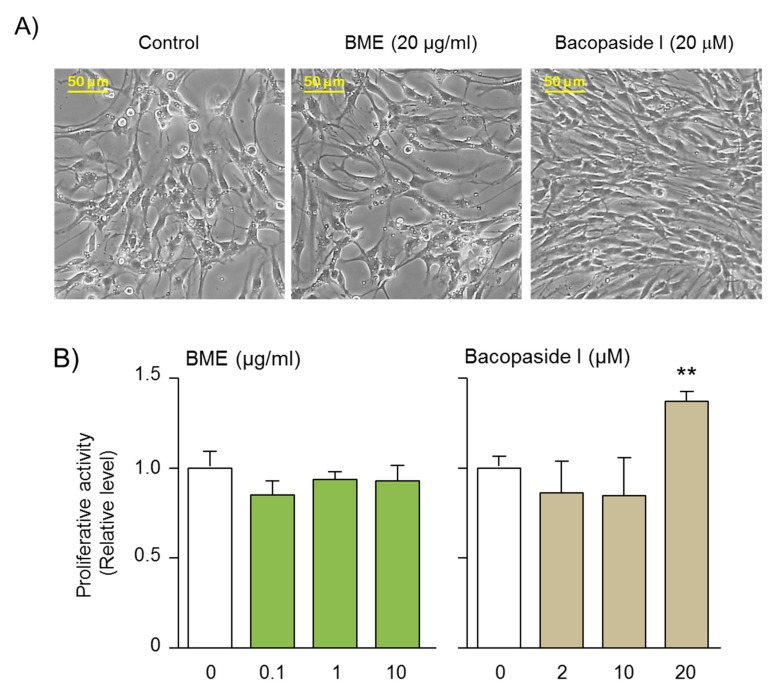
Effect of BME and bacopaside I, a major triterpenoid constituent of BME, on the proliferation of the NPCs. Cells were prepared from hippocampal dentate gyrus, as described in the Material and Methods section. After cultivating for 14–20 days, NPCs were harvested for subsequent replating and culturing in the growth medium in the absence or presence of different concentrations of BME and bacopaside I for assessment of cell proliferation by 3-(4,5-Dimethylthiazol-2-yl)-2,5-diphenyltetrazolium bromide (MTT) assay. (**A**) Typical images of NPCs cultured in the absence or presence of BME (20 μg/ml) and bacopaside I (20 μM). (**B**) Summarized data on NPC proliferation after incubation for 6 days with indicated treatments. Each data column represents the mean ± SEM from 4 separate experiments. ** *p* < 0.01, significantly different from the non-treated group.

Our previous study evaluated the effects of isolated bacosides, including bacopaside I, bacopaside N2, and a mixture of bacopaside II and bacosaponin D, against oxygen- and glucose deprivation (OGD)-induced neuronal cell damage in organotypic hippocampal slice cultures (OHSCs) (Figure S1) [8]. The results demonstrated that only bacopaside I promoted neuroprotection against OGD-induced hippocampal cell damage, and this occurred via protein kinase C (PKC) and phosphatidylinositol-3 kinase (PI3K)/Akt mechanisms (Figure S2) [8]. Based on these findings, in this study, we asked whether BME and bacopaside I affect the proliferation of neural progenitor cells (NPCs) that are responsible for neurogenesis in the hippocampus. The results showed that the treatment of cultured NPCs with bacopaside I (20 μM) for 6 days significantly enhanced the proliferative activity of NPCs (Figure 5). However, BME treatment at concentrations as high as 10 μg/mL had no significant effect on the proliferation of NPCs, and higher amounts of BME caused toxicity. As the concentration of bacopaside 1 in BME is only 11%, its level is too low to replicate the effect of purified 20 μM bacopaside 1; however, this result indicates that other bioactive components are not present at high concentrations.

**Figure 6:**
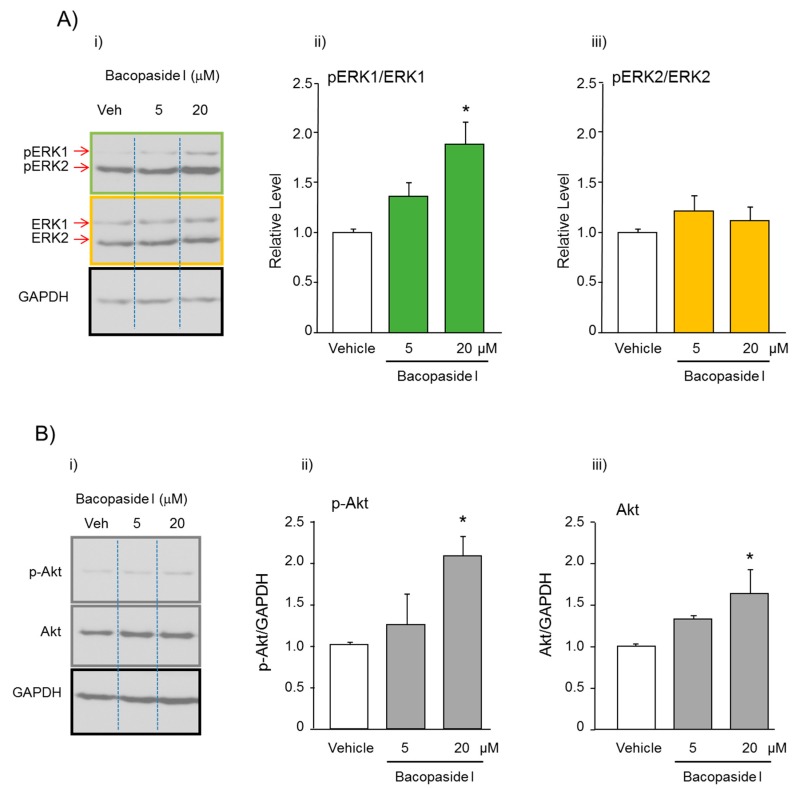
Effect of bacopaside I on the expression levels of proteins related to proliferation in cultured neural progenitor cells (NPCs) derived from the dentate gyrus. NPCs were incubated with vehicle or bacopaside I (5 and 20 μM) for 24 h. (**A**) i: Typical photos indicating the expression levels of p-Erk1, p-Erk2, Erk, Erk2, and GAPDH. ii and iii: Quantitative comparisons of changes in the expression ratios between phosphorylated and non-phosphorylated proteins. (**B**) i: Typical photos indicating the expression levels of p-Akt, Akt, and GAPDH. ii and iii: Quantitative comparisons of the levels of each protein in the NPCs. The expression levels of GAPDH were used to normalize the signals between various samples. Each data column represents the mean ± S.E.M. (*n* = 4). * *p* < 0.05. versus vehicle group.

To study the molecular mechanisms underlying bacopaside I- induced proliferation in NPC cultures, the expression levels of ERK1/2 (extracellular signal-regulated kinase 1/2) and Akt (Protein kinase B-PKB) were analyzed by western blotting. Treatment of NPCs with bacopaside I (5 and 20 μM) for 24 hr significantly increased the expression ratio between pERK1 and ERK1 (F(2,9) = 11.530, *p* = 0.003), while it did not affect the expression ratio between pERK2 and ERK2 (F(2,9) = 0.987, *p* = 0.410). Moreover, the same treatment also significantly increased the expression levels of total Akt (F(2,9) = 5.166, *p* = 0.032) and phospho-Akt (p-Akt, Ser473), an active form of Akt (F(2,9) = 4.926, *p* = 0.036), in a concentration-dependent manner. The expression levels of Akt and p-Akt were increased about 60 and 100%, respectively, by treatment with 20 μM bacopaside I (Figure 6).

## 3. Discussion

The present study investigated the effects of daily BME treatment (50 mg/kg/day) on spatial working memory performance of adolescent 5-week-old and adult 8-week-old mice. The results demonstrated that the administration of BME daily to adolescent mice enhanced spatial cognitive function and correlated with the promotion of endogenous neurogenesis in the hippocampal dentate gyrus region. Furthermore, our in-vitro experiments using an NPC culture system suggested that bacopaside I, a major triterpenoid constituent of BME, contributes to the effect of BME on the hippocampal neurogenesis by enhancing proliferation of NPCs.

In this study, we employed a modified version of the Y-maze test to elucidate the spatial working memory of subject mice because this paradigm can be repeated with the same animal groups, when separated by intervals of no less than one week [10,18]. When 5-week-old mice were treated daily with BME for 7–28 days from day 0, significant enhancement of the spatial cognitive function in the modified Y-maze test occurred on day 7 but not on day 28. Notably, these results are consistent with clinical studies showing that supplementation with a combination of BME and multiple micronutrients to school children aged 7–12 years for 60 days but not 121 days significantly improved spatial working memory [19]. Furthermore, the treatment of neonatal rats with BME caused improvement in spatial memory performance and memory retention at 2–6 weeks after starting the administration [20]. Mice at the age of 5 and 8 weeks are adolescent and adult, respectively [21,22,23,24], and development of cognitive and physical function during adolescence is susceptible to training, drugs, nutrition exposure, or other environmental factors [25]. Together, the present results allow us to hypothesize that the enhancing effect of BME on spatial cognitive function depends on the age of mice used and becomes evident only when the animals are treated during adolescence.

This hypothesis is supported by the results obtained with three different experimental protocols. As shown in Figure 3, the 5-week-old adolescent mice treated with BME daily for 7 days showed enhanced cognitive performance compared to the same animal group treated with vehicle water. In contrast, the 8-week old adult mice did not responde to the BME treatment and showed no difference in cognitive performance from the vehicle-treated adult animals. Moreover, it is of interest to note that the enhancement of working memory performance by BME administration during adolescence was maintained even 3 weeks after terminating the BME treatment. These results suggest that repeated treatment with BME during adolescence may be essential for long-lasting enhancement of cognitive function in mice.

One may infer that the failure to improve cognitive performance in mice treated with BME for 4 weeks was due to the BME-induced toxicity. However, this possibility is unlikely because our preliminary study showed that BME at doses up to 1.77 g/kg failed to induce any acute toxic symptoms in mice. Moreover, in agreement with other reports [26], daily treatment of rats with BME at a dose equivalent to the pharmacological effects in mice (50 mg/kg) for 30 days induced no mortality or adverse effects on the biological and morphological indices, including body weight, relative organ weights, hematological, and biochemical parameters, histopathology of liver and kidneys.

To have a better understanding of the mechanism by which the administration of BME during adolescence enhanced the spatial cognitive function, we conducted an immunohistochemical analysis of neurogenesis in the hippocampus. The results revealed that the treatment of adolescent mice with BME daily for 7 days significantly enhanced cell proliferation in the dentate gyrus region without affecting cell death. Besides, a significant increase in the hippocampal neurogenesis persisted even 3 weeks after terminating the BME administration. Evidence indicates that promotion and suppression of hippocampal neurogenesis are associated with improvement and impairment in learning and memory performances, respectively [6,7]. Indeed, our previous study using a trimethyltin (TMT)-induced neurotoxicity model of mice demonstrated that the daily administration of BME improved cognitive deficits caused and that the effects of BME were due to not only protection of hippocampal cell from TMT toxicity but also facilitation of hippocampal neurogenesis [15]. Given these findings, the present results suggest that the daily administration of BME during adolescence is essential to elicit long-term promotion of a neurogenesis process in the dentate gyrus. This enhancement of cognitive function in adolescent animals lasts even after the animals reached adulthood.

The RNAseq analysis demonstrated several noteworthy features of treatment with BME. Those are, first, a significant decrease in the expression of gene coding PP2A, a modulator of the PI3K/Akt pathway that is involved in neuronal signaling and neurodevelopment [27], and second, changes in the expression of genes coding Wnt, neurogenin, Igf, VEGF, ChAT, and CREB, that are involved in hippocampal neurogenesis and cognitive function in rodents [28,29,30,31,32,33,34]. For example, the activation of Wnt signaling pathway reportedly enhances the cognitive function of adult mice and rescues memory deficit in APP/PS1-transgenic mice, an Alzheimer’s disease model [35], whereas blockade of Wnt signaling reduces hippocampal neurogenesis [28]. Moreover, our previous study demonstrated possible involvement of ChAT and CREB in the mechanism by which BME treatment enhanced cognitive function in OBX mice [10]. Taken together, the data obtained by the RNAseq analysis provide mechanistic evidence for BME treatment-induced enhancement of cognitive function and neurogenesis in the hippocampus.

Finally, we conducted in vitro experiments using NPCs to elucidate the role of bacopaside I in BME-induced promotion of neurogenesis in adolescent animals. We employed bacopaside I, a major triterpenoid component of BME, because our previous study using a brain ischemia model suggested that bacopaside I (25 μM) has a major role in the neuroprotective effects of BME in this animal model via PKC and PI3K/Akt pathways [8]. Bacopaside I is also reported to enhance the BDNF signaling pathway in a mouse model of depression, at least in part via activation of pERK/pCREB/BDNF signaling [36] involved in the neurotrophic activity and neurogenesis [37,38]. In agreement with these reports, we found that bacopaside I at 20 μM can promote the proliferation of NPCs probably via ERK1/2 and Akt mechanisms. ERK1/2, a subclass of mitogen-activated protein kinases (MAPKs), are involved in the growth, proliferation, and survival of various types of cells, including NPCs [39,40]. The present results showed that bacopaside I dose-dependently enhanced the phosphorylation of ERK1 but not the phosphorylation of ERK2.

It is also of interest that bacopaside I, at 20 μM, induced a significant increase in the expression levels of Akt and p-Akt in NPCs. This finding is supported by the RNAseq analysis, in which we demonstrated that BME enhanced the expression of Akt3 mRNA in the hippocampus. Akt3 plays an important role in cell proliferation [41]. In addition, the extent of the increase in phospho-Akt was more than the extent of the increase in non-phosphorylated Akt in the bacopaside I-treated NPCs, suggesting that bacopaside I enhances not only translation of Akt protein from its mRNA but also the PI3K/Akt signaling pathway in NPCs. Several lines of evidence indicate that the PI3K/Akt pathway controls the proliferation, differentiation, and migration of endogenous neural stem cells, and plays a crucial role in neurogenesis [42,43]. In conclusion, the present study demonstrates that BME enhances working memory performance in adolescent mice by promoting hippocampal neurogenesis and that the effects of BME are due in significant amounts, to bacopaside I.

## 4. Materials and Methods

### 4.1. Preparation of BME

*The Bacopa monnieri* (L.) Wettst. (BM) used in the present study was that used in previous studies [8,15]. It was collected in Ho Chi Minh city, Vietnam and identified by Dr. Pham Thanh Huyen, Department of Resource Medicinal Material, National Institute of Medicinal Material, Hanoi, Vietnam (NIMM) and submitted as voucher specimen 9967 in NIMM. BME was prepared as previously described [8,15]. Briefly, BM aerial parts were dried at 50 °C, cut into small pieces, crushed and extracted with 50% ethanol (1:8 *w*/*v*) at 85 °C for 2 h, twice filtered through filter paper and concentrated under reduced pressure at 50 °C. After further extraction three times with n-butanol, the extract was subjected to chemical analysis using a high-performance liquid chromatographic system (HPLC; Shimazu, Japan) [8] and determined to contain 21.8% bacoside A and 11.0% bacopaside I. Bacopaside I, a triterpene saponin of BM, was isolated by Dr. Nguyen Van Tai, Department of Phytochemistry, National Institute of Medicinal Materials, Vietnam, as previously described [8] and determined to be ≥ 95% by HPLC-MS analysis. (Mass spectrometry data is available at http://wakandb.u-toyama.ac.jp/wiki/ LCMS: Bacopa_INM-484).

### 4.2. Animals

**Figure 7:**
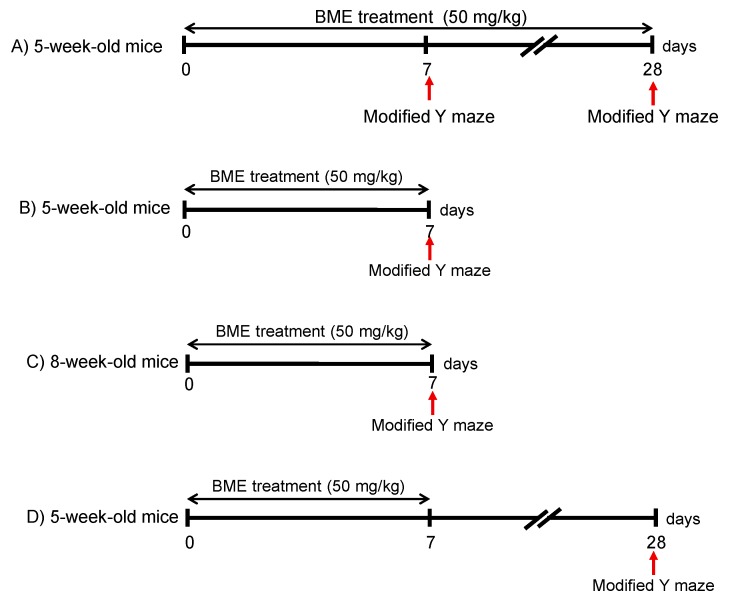
Experimental schedule to examine the effect of BME (50 mg/kg) on spatial working memory in adolescent (5-week-old) or adult (8-week-old) mice using the modified Y maze. (**A**): Adolescent mice were orally administered BME for 28 days. The spatial cognitive performance of the animals was elucidated at days 7 and 28 after the first BME treatment. (**B**) and (**C**): Adolescent (**B**) or adult mice (**C**) mice were orally administered BME for 7 days, respectively, and then a modified Y maze test was conducted at day 7 after the first BME treatment. (**D**): Adolescent mice were orally administered BME for 7 days, and a modified Y maze test was conducted at day 28 after the first BME treatment.

Male Swiss albino mice, 4 weeks old (adolescent mice) or 7 weeks old (adult mice), were obtained from the National Institute of Hygiene and Epidemiology, Hanoi, Vietnam, and used for behavioral experiments and RNAseq analysis. Four-weeks-old ddY mice (Japan, SLC, Shizuoka, Japan) were used for histochemical analysis and cultured neural progenitor cells. The animals were habituated to the animal rooms for one week before the start of the experiments and given food and water ad libitum. Housing was maintained at 24 ± 1 °C with 65 ± 5% humidity and a 12-hr light-dark cycle (lights on: 07:00–19:00). Behavioral experiments were performed during the light phase from 9:00 to 18:00, per the experimental schedules described in Figure 7. Experimental protocols were approved by the Institutional Animal Use and Care Committees of the National Institute of Medicinal Materials (NIMM), Hanoi, Vietnam and the Committee for the Ethical Use of Experimental Animals, Setsunan University, Japan.

### 4.3. Behavioral Study4.3. Modified Y Maze Test

Mice were randomly divided into two groups: vehicle (water)-treated mice (control) and BME-treated mice (BME group). BME (50 mg/kg) was dissolved in distilled water and orally administered for 7 or 28 days as described previously [8,10,15]. A modified Y maze test was conducted as described previously [8,15,18] at day 7 and 28 after the first BME treatment (Figure 7).

The apparatus used for the test consisted of black polypropylene walls with three arms, each 40 cm long, 12 cm wide at the top, 3 cm wide at the bottom, and 18 cm high. The test comprised a sample trial and a test trial separated by an interval; in the sample trial, each mouse was placed in the maze with one of the three arms closed. The animal was allowed to explore the other two arms freely for five min, then removed. Thirty minutes after the sample trial, the animal was again placed in the maze with all three arms open and allowed to explore freely. The previously closed arm is defined as the novel arm. The animal behavior was video-recorded using Any Maze system^®^ (Stoelting Co, IL, USA), and the percent time spent in the novel arm and numbers of total arm entries were analyzed as previously described [8,15,18].

### 4.4. Immunohistochemistry

Immunohistochemical studies were conducted as previously described [15,44]. The ddY male adolescent mice received injections of BrdU (50 mg/kg, i.p.) two times at a 12 hr interval to label mitotic cells. The daily administration of BME (50 mg/kg, p.o.) or vehicle water was started immediately after the first BrdU injection (day 0) and continued until day 7.

#### 4.4.1. Preparation of Hippocampal Slices

On days 7 and 28 after first BME treatment, mice were decapitated under chloral hydrate anesthesia (500 mg/kg, i.p.) and fixed by intracardiac perfusion with 0.1M phosphate-buffered saline (PBS, pH 7.4) followed by 4% (*w*/*v*) paraformaldehyde in PBS. The brain tissues were dissected and further fixed with the same solution at 4 °C overnight. Post-fixed brains were embedded in paraffin and sagittal sections with a thickness of 3–5 µm which were prepared at 100-µm intervals in a range between 0.9 and 1.6 mm relative to the lateral, according to the atlas of Paxinos and Franklin [45]. The paraffin-embedded brain sections were then deparaffinized with xylene, rehydrated by immersion in ethanol of graded decreasing concentrations of 100% to 50% (vol./vol.), and washed with water.

#### 4.4.2. Immunostaining

Immunostaining was conducted using the brain tissues obtained on days 7 and 28. According to the methods previously reported [15,44,46], newly generated cells, neuronal cell death, and neuronal cells were determined by staining BrdU-, FJB-, and NeuN-positive cells, respectively. Stained sections were viewed with a BX41 microscope (Olympus, Tokyo, Japan) equipped with a DS-Ri1 camera (Nikon, Tokyo, Japan), and the number of labeled cells was counted via microscopic observation. All seven sagittal sections prepared from the brain of each mouse were used for immunostaining and counting of positive cells in the whole dentate gyrus (granule cell layer-GCL, subgranular zone-SGZ, hilus, molecular layer) and GCL ± SGZ.

### 4.5. RNA Sequence Analysis

Adolescent Swiss albino mice (5-week-old, *n* = 6/group) were treated with BME (50 mg/kg, p.o) daily from day 1 for one week. On day 7, 1 hr after BME treatment, animals were euthanized by chloral hydrate anesthesia (500 mg/kg, i.p.) to dissect hippocampi. Total RNA from the hippocampal tissues was extracted using Trisure^TM^ (Bioline Reagents Ltd. United Kingdom) according to the manufacturer’s instructions. DNA libraries were prepared and sequenced on an Illumina NextSeq 500™ at the University of Missouri DNA Core. The quality-controlled RNASeq reads (approximately 20 million/sample) were aligned to the reference genome of *Mus musculus* as described by Pertea et al. [47]. The genes categorized as involved in “neurogenesis and/or memory” by gene functional annotation with a cutoff value of multimodal *p* < 0.05 and fold change of > 2 or <−2 were identified as being differentially expressed, and data reformatted, sorted and filtered using DAVID (https://david.ncifcrf.gov/). The analysis revealed significantly altered expression of genes for neuronal signaling pathways for cell survival and replication, using a cutoff value of multimodal *p* < 0.05 and fold change > 1.2 or < −1.2. Gene functions were identified by using Genecards (https://www.genecards.org) and/or Mouse Genome Databases (http://www.informatics.jax.org).

### 4.6. In-Vitro Neural Progenitor Cell (NPCs) Culture and Proliferation Assay

#### 4.6.1. NPC Culture and MTT Assay

Cultures of NPCs were prepared from the hippocampal dentate gyrus of adolescent mice under microscopic observation [48]. The cells were suspended in 3 mL of Neurobasal A medium (NAM: Life Technologies/Gibco, Eugene, OR) by pipetting, and then centrifuged at 200g for 5 min. The pellets were suspended with 4 mL of NAM containing 2 mg/mL papain, 0.5 mg/mL deoxyribonuclease, and 0.18 mg/mL neutral protease and then incubated at 37 °C for 4 min. The cell suspensions were mixed with an equal volume of NAM and centrifuged at 200g for 5 min. The pellets were suspended with 5.0 mL of Percoll buffer solution consisting of NAM (3.9 mL), Percoll (0.99 mL), and 10XPBS (0.11 mL) and centrifuged at 400 g for 15 min. The pellets were washed three times with 4 mL of NAM and cells were suspended in 2 mL of growth medium consisting of Neurobasal A medium containing B27 supplement, 2 mM Glutamax, 100 U/mL penicillin, 0.1 mg/mL streptomycin, 20 ng/mL epidermal growth factor, and 20 ng/mL basic fibroblast growth factor. The surfaces of the culture dishes were coated at 37 °C for 2 h with solution containing 15 µg/mL poly-l-ornithine and 5 µg/mL laminin. The cells were then seeded at a density of 3 × 10^4^ into six-well dishes (Greiner Bio-One, Frickenhausen, Germany) then cultured for 24 h in the growth medium, which was replaced with fresh medium on the next day. Subsequently, the cultures were maintained by feeding with 75% replacement of the growth medium every 2 days for 14–20 days in vitro to prepare primary cultures of NPCs. For secondary cultures of NPCs, the primary cultures were harvested, dispersed, replaced at a density of 3 × 10^4^ cells/mL into six-well dishes, and incubated for 6–8 days at 37 °C in a 5% CO_2_/95% air-humidified incubator. Finally, the secondary cultures were harvested, dispersed, and then replaced at a density of 3 × 10^4^ cells/mL in six-well dishes as tertiary cultures, which were used for the experiments. Cells were cultured in the growth medium in the absence or presence of of BME (0.1, 1 and 10 µg/mL), and bacopaside I (2, 10 and 20 µM) for 6 days at 37°C in a 5% CO_2_/95% air-humidified incubator. Viability of NPC was determined by the MTT assay as previously described [49].

#### 4.6.2. Western Blotting

The secondary cultures of NPCs were cultured at a density of 20 × 10^4^ cells/mL in six-well dishes. Cells were exposed to bacopaside I (5 and 20 µM) at 37 °C in a 5% CO_2_/95% air-humidified incubators. After 24 h, the cells were harvested for Western blotting analysis. The protocols for protein determination and Western blotting have been described [48]. Briefly, total protein (15 μg) prepared from each sample was separated by 10% SDS-polyacrylamide gel (SDS-PAGE) and transferred to polyvinylidene difluoride membrane (Immun-Blots Membrane for Protein Blotting, Bio-rad Laboratory, CA, USA). The membranes were probed with the following primary antibodies: anti-p44/42 MAPK (Erk1/2) rabbit polyclonal antibody (1:3000 dilution); anti-phosphor-p44/42 MAPK (Erk1/2) (Thr202/Tyr204) rabbit polyclonal antibody (1:3000 dilution); anti-Akt rabbit monoclonal antibody (1:2000 dilution); phosho-Akt rabbit monoclonal (p-Akt, Ser743) antibody (1:1000 dilution) (Cell Signaling Technology, Danvers, MA, USA); or anti-GAPDH rabbit monoclonal antibody (1:1000 dilution, Santa Cruz Biotechnology, Santa Cruz, CA, USA) at 4 °C overnight. Afterward, the membranes were further treated with horseradish-peroxidase-labeled secondary antibodies (1:3000 dilution, DakoCytomation EnVision System-HRP Labeled Polymer, CA, USA) at room temperature for 1 hr. The expression levels of each signal were detected with the aid of Western Lightning Chemoluminescence Reagent Plus and exposure to X-ray films. The membranes were reprobed using a Blot Restore Membrane Rejuvenation Kit (Millipore, Temecula, CA, USA). The density of immunoreactive bands was analyzed using CS Analyzer (Atto Co.).

### 4.7. Statistical Analysis

Data were expressed as the mean ± SEM. All of the data obtained in the present study, except those from the cultured neural progenitor cells, were analyzed by Student’s *t*-test or Welch’s *t*-test. Cultured neural progenitor cells were analyzed using one-way ANOVA followed with post hoc Dunnett’s test using SPSS version 23.0. Differences of P < 0.05 were considered significant.

## Figures and Tables

**Table 1:** BME treatment-induced changes in the expression levels of genes involved in neurogenesis-related signaling pathways in the hippocampus. The down-regulation of genes is indicated by the values in brackets. The function of each gene was identified by Genecards (https://www.genecards.org) and/or Mouse Genome Databases (http://www.informatics.jax.org).

Signaling Pathway	Gene Products	Gene Symbol	Fold Change	*p*-Value	Neurogenesis-Related Function Citation
Neurotrophin signaling pathway	NT3	NTRK2 (TrKB)	1.325	< 0.0001	Receptor tyrosine kinase is involved in the development and the maturation of the central and the peripheral nervous systems through regulation of neuron survival, proliferation, migration, differentiation, and synapse formation and plasticity. Receptor for BDNF/brain-derived neurotrophic factor and NTF4/neurotrophin-4, alternatively, can also bind NTF3/neurotrophin-3, which is less efficient in activating the receptor but regulates neuron survival.
		NTRK3 (TrKC)	1.501	< 0.0001	NTF3/neurotrophin-3, NTRK3, autophosphorylates and activates different signaling pathways, including the phosphatidylinositol 3-kinase/AKT and the MAPK pathways, which control cell survival and differentiation.
PI3K-Akt	PP2A	Ppp2r3d	(1.342)	0.005	Cell proliferation
		Ppp2r1a	(1.371)	0.012	Cell death, cell differentiation, cellular component organization
		Ppp2r5a	(1.228)	0.009	Lipid metabolic process, protein metabolic process, response to stimulus, signaling
		Ppp2r5b	(1.354)	0.008	Cell differentiation, cellular component organization, nucleic-acid-templated transcription, protein metabolic process response to stimulus, signaling, system development
	PI3K class IA	Pik3ca	1.342	0.009	Cell death, cell population proliferation, establishment of localization, homeostatic process, lipid metabolic process, protein metabolic process, response to stimulus signaling, system development
		Pik3cb	1.351	0.001	Cell death, establishment of localization, homeostatic process, immune system process, lipid metabolic process, response to stimulus, signaling, system development
		Pik3r1	1.367	0.008	Cell death, cell differentiation, cell population proliferation, cellular component organization, establishment of localization, homeostatic process, immune system process, lipid metabolic process, nucleic-acid-templated transcription, protein metabolic process, response to stimulus, signaling, system development
		Pik3cg	1.968	0.000	Cell death, establishment of localization, homeostatic process, immune system process, lipid metabolic process, protein metabolic process, response to stimulus signaling, system development
	AKT	Akt1	(1.282)	0.035	Cell death, cell differentiation, cell population proliferation, cellular component organization, establishment of localization, homeostatic process immune system process, lipid metabolic process, nucleic-acid-templated transcription, protein metabolic process response to stimulus, signaling, system development
		Akt3	1.615	< 0.0001	Cell population proliferation, cellular component organization, establishment of localization, homeostatic process
	GF	HgfP	2.213	0.035	Cell death, cell differentiation, cell population proliferation, cellular component organization
	PKC	PrkcaR	1.546	< 0.0001	Cell death, cell differentiation, cell population proliferation, cellular component organization, establishment of localization, homeostatic process, immune system process
		Prkcb	1.249	0.000	Cell death, cellular component organization, establishment of localization, homeostatic process, immune system process, nucleic-acid-templated transcription protein metabolic process, response to stimulus signaling system development
CREB	pCREB	Creb1	1.337	0.016	Cell differentiation, cellular component organization, signaling, system development
		Atf2	1.323	0.003	Cell death, cell differentiation, cell population proliferation, cellular component organization, establishment of localization
Cholinergic receptor	ChAT	ChatE	3.970	0.005	Cell differentiation, cellular component organization, signaling, system development
	M2	Chrm2	1.582	0.003	Establishment of localization, response to stimulus, signaling
	M5	Chrm5	2.592	0.000	Establishment of localization, lipid metabolic process, response to stimulus, signaling

## Supplementary Materials

Supplementary materials can be found at https://www.mdpi.com/1422-0067/21/9/3365/s1.

## Abbreviations

BME	*Bacopa monnieri* L. Wettst. extract
TMT	Trimethyltin
GCL	Granule cell layer
SGZ	Subgranular zone
PKC	Protein kinase C
PI3K	Phosphatidylinositol-3 kinase
NPC	Neural progenitor cells
FJB	Fluoro-Jade B
NAM	Neurobasal A medium
BrdU	5-Bromo-2´-Deoxyuridine
NeuN	Neuronal nuclei
OBX	Olfactory bulbectomized
ChAT	Choline acetyltransferase
PI3K/AKT	Phosphoinositide 3-kinases/ Protein kinase B
pAkt	Phophorylated protein kinase B (Akt)
ERK1/2	Extracellular signal-regulated kinase 1/2
pERK1/2	Phophorylated extracellular signal-regulated kinase 1/2
PPP2A	Protein Phosphatase 2A
CREB	cAMP response element-binding protein
BDNF	Brain-derived neurotrophic factor
NAM	Neurobasal A medium
MTT	3-(4,5-Dimethylthiazol-2-yl)-2,5-diphenyltetrazolium bromide
