# Supplementary material for: Bacopa monnieri (L.) Wettst. Extract Improves Memory Performance via Promotion of Neurogenesis in the Hippocampal Dentate Gyrus of Adolescent Mice
Source: Int J Mol Sci. 2020 May 9;21(9):3365. doi: 10.3390/ijms21093365 (PMC7247711; doi:10.3390/ijms21093365)
Supplement: Supplementary file 1 [file ijms-21-03365-s001.pdf]

Supplementary Table 1. The 7-day treatment with BME (50 mg/kg)-induced changes in the expression levels of genes involved in neurogenesis and/or cognitive function in adolescent mice. A cutoff value of multimodal $P < 0.05$ and fold-change > 2 or < -2 were set. The fold change of down-regulation of genes was indicated by the values in brackets. The RNA-seq results were analyzed by using David software from 6 mice per group. Gene functions were identified by using Genecards database (https://www.genecards.org).

Gene symbol	Gene name	Fold change	Function
Adnp	Activity-dependent neuroprotective protein	2.12	Potential transcription factor. May mediate some of the neuroprotective peptide VIP-associated effects involving normal growth and cancer proliferation. When isolated from the sequence, neuroprotective peptide provides neuroprotection against the amyloid-beta peptide.
Aff2	AF4/FMR2 family, member 2	2.32	RNA-binding protein. Might be involved in alternative splicing regulation through an interaction with G-quartet RNA structure. Play a role in brain development and learning or memory.
Barhl2	BarH-like 2 (Drosophila)	3.22	Potential regulator of neural basic helix-loop-helix genes. GOBP indicated that gen play a role in nervous system development and neuron migration.
Ccl5	Chemokine (C-C motif) ligand 5	(2.51)	May be an agonist of the G protein-coupled receptor GPR75, stimulating inositol trisphosphate production and calcium mobilization through its activation. May play a role in neuron survival through activation of a downstream signaling pathway involving the PI3, Akt and MAP kinases.
Chat	Choline acetyltransferase	3.97	Catalyzes the reversible synthesis of acetylcholine (ACh) from acetyl CoA and choline at cholinergic synapses; phosphatidylcholine biosynthetic process, neurotransmitter secretion, neuromuscular synaptic transmission, and acetylcholine biosynthetic process and neurotransmitter biosynthetic process.
Crh	Corticotropin releasing hormone	(2.26)	Positive regulation of protein phosphorylation; synaptic transmission; dopaminergic; positive regulation of protein phosphorylation.

FERD3L	Fer3-like (Drosophila)	5.56	Regulation of neurogenesis; cell development
Foxo6	Forkhead box O6 (Foxo6)	(2.03)	Transcriptional activator; memory
Grpr	Gastrin releasing peptide receptor	3.86	Learning or memory; neuropeptide signaling pathway; phospholipase C-activating G protein-coupled receptor signaling pathway; G protein-coupled receptor signaling pathway, signal transduction.
Gsx1	GS homeobox 1	(2.31)	Spinal cord association neuron differentiation; central nervous system development
Id3	Inhibitor of DNA binding 3	(2.04)	Multicellular organism development; transcription, DNA-templated
Ier2	Immediate early response 2	(2.25)	Neuron differentiation; cell motility; response to fibroblast growth factor
Igf2	Insulin-like growth factor 2	2.43	Negative regulation of transcription by RNA polymerase II; skeletal system development; ossification; osteoblast differentiation; in utero embryonic development.
Il1b	Interleukin 1 beta	(2.09)	Activation of MAPK activity; positive regulation of protein phosphorylation; positive regulation of T cell mediated immunity.
Il1rn	Interleukin 1 receptor antagonist	6.68	Lipid metabolic process; inflammatory response; immune response; signal transduction; cytokine-mediated signaling pathway.
Isl1	ISL1 transcription factor, LIM/homeodomain	5.14	Negative regulation of transcription by RNA polymerase II; neural crest cell migration; outflow tract septum morphogenesis.
Lhx5	LIM homeobox protein 5	2.36	Regulation of transcription, DNA-templated; cell-cell signaling; spinal cord association neuron differentiation; cerebellum development; cerebellar Purkinje cell differentiation.
Lhx8	LIM homeobox protein 8	15.76	Transcription factor involved in differentiation of certain neurons and mesenchymal cells.
Musk	Muscle, skeletal, receptor tyrosine kinase	4.14	Receptor tyrosine kinase which plays a central role in the formation and the maintenance of the neuromuscular junction (NMJ), the synapse between the motor neuron and the skeletal muscle. May also play a role within the central nervous system by mediating cholinergic responses, synaptic

			plasticity and memory formation.
Neurod4	Neurogenic differentiation 4	2.53	Neuron migration; Notch signaling pathway; multicellular organism development
Neurog1	Neurogenin 1	(4.28)	Acts as a transcriptional regulator. Involved in the initiation of neuronal differentiation. Associates with chromatin to enhancer regulatory elements in genes encoding key transcriptional regulators of neurogenesis.
Oprm1	Opioid receptor, mu 1	3.15	Acute inflammatory response to antigenic stimulus; adenylate cyclase-activating dopamine receptor signaling pathway; G protein-coupled receptor signaling pathway.
Otx2	Orthodenticle homeobox 2	2.20	Regulation of transcription, DNA-templated; axon guidance; forebrain development
Oxt	Oxytocin	(2.63)	Signal transduction; G protein-coupled receptor signaling pathway.
Prdm12	PR domain containing 12	3.37	Neurogenesis; neuron projection development
Shc3	Src homology 2 domain-containing transforming protein C3	2.66	MAPK cascade, Ras protein signal transduction
Sox3	SRY (sex determining region Y)-box 3	(2.53)	Regulation of transcription by RNA polymerase II; multicellular organism development; central nervous system development
Trpc5	Transient receptor potential cation channel, subfamily C, member 5	2.81	Nervous system development; positive regulation of cytosolic calcium ion concentration
Wnt6	Wingless-type MMTV integration site family, member 6	(2.04)	Multicellular organism development; positive regulation of gene expression; Wnt signaling pathway

Supplementary Fig. 1 (Fig. 3 Le et al, 2015)

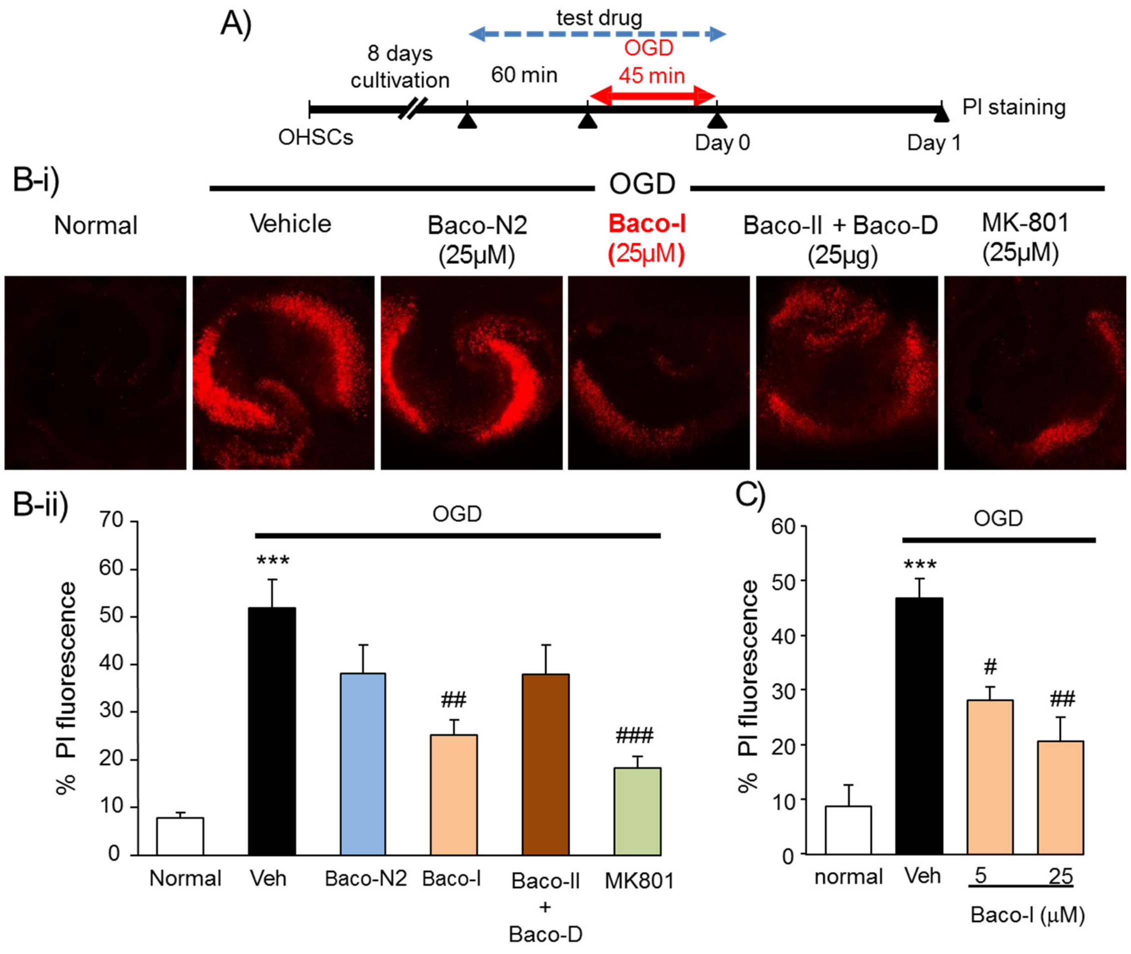

Supplementary Fig. 1. Effects of bacosides on OGD-induced neuronal cell damage in OHSCs. **(A)** Experimental protocol. OHSCs were

treated with test drugs for 60 min before OGD and during the 45-min period of OGD. Neuronal cell damage was evaluated by measuring the PI uptake signal 1 day after the OGD challenge. **(B-i)** Typical PI staining images of OHSCs treated with 45-min OGD in the absence (vehicle) and presence of bacopaside N2 (Baco-N2: 25μM), bacopaside I (Baco-1: 25μM), a mixture of bacopaside II (Baco-II) and bacosaponin D (Baco-D: 25μg/ml), and the NMDA receptor antagonist MK-801 (25μM). **(B-ii)** Summarized data obtained from (B-i). (C) A dose-dependent effect of Baco-I. Each data column represents the mean ± S.E.M (n=5-6). ***p<0.001 vs. normal OHSCs; #p<0.05, ##p<0.01, and ###p<0.001 vs. OHSCs treated with OGD alone.

Supplementary Fig. 2 (Fig. 7 Le et al, 2015)

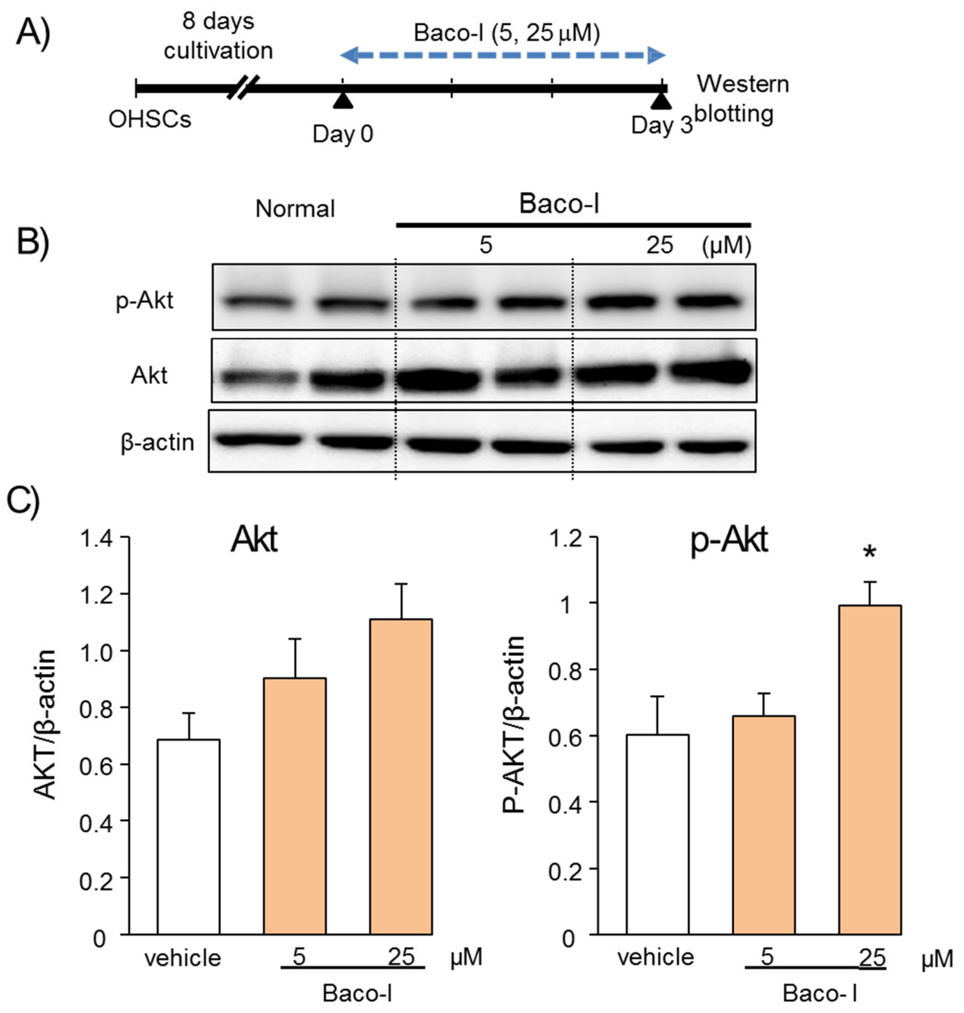

Supplementary Fig. 2. Bacopaside I-induced increases in the expression level of p-Akt in OHSCs. (A) Experimental protocol. OHSCs were incubated with 5 and 25 μM bacopaside I (Baco-I) for 3 days before Western blotting analysis. (B) Typical photos indicating the expression levels of Akt and p-Akt (Ser473) in the OHSCs. (C) Quantitative comparisons of the expression levels of Akt and p-Akt in OHSCs. Each data column represents the mean ± S.E.M (n=4). *p<0.05 vs. vehicle-treated OHSCs.

Reference

Le, X.T.; Nguyet Pham, H.T.; Van Nguyen, T.; Minh Nguyen, K.; Tanaka, K.; Fujiwara, H.; Matsumoto, K. Protective effects of Bacopa monnieri on ischemia-induced cognitive deficits in mice: the possible contribution of bacopaside I and underlying mechanism. *Journal of ethnopharmacology* 2015, *164*, 37-45, doi:10.1016/j.jep.2015.01.041.
